# Invertebrate genetic models of amyotrophic lateral sclerosis

**DOI:** 10.3389/fnmol.2024.1328578

**Published:** 2024-03-04

**Authors:** LiJun Zhou, RenShi Xu

**Affiliations:** ^1^Department of Neurology, National Regional Center for Neurological Diseases, Clinical College of Nanchang Medical College, Jiangxi Provincial People's Hospital, First Affiliated Hospital of Nanchang Medical College, Xiangya Hospital of Central South University Jiangxi Hospital, Nanchang, Jiangxi, China; ^2^Medical College of Nanchang University, Nanchang, China

**Keywords:** amyotrophic lateral sclerosis, invertebrate models, yeast, *Drosophila melanogaster*, *Caenorhabditis elegans*, *SOD1*, *TDP-4*3, *FUS*

## Abstract

Amyotrophic lateral sclerosis (ALS) is a common adult-onset neurodegenerative disease characterized by the progressive death of motor neurons in the cerebral cortex, brain stem, and spinal cord. The exact mechanisms underlying the pathogenesis of ALS remain unclear. The current consensus regarding the pathogenesis of ALS suggests that the interaction between genetic susceptibility and harmful environmental factors is a promising cause of ALS onset. The investigation of putative harmful environmental factors has been the subject of several ongoing studies, but the use of transgenic animal models to study ALS has provided valuable information on the onset of ALS. Here, we review the current common invertebrate genetic models used to study the pathology, pathophysiology, and pathogenesis of ALS. The considerations of the usage, advantages, disadvantages, costs, and availability of each invertebrate model will also be discussed.

## 1 Introduction

Amyotrophic lateral sclerosis (ALS) is a multifactorial non-cell-autonomous neurodegenerative disease. Since the 1990s, several genes and mutations have been found to be involved in the pathogenesis of ALS. Based on this, several genetic animal models have been developed to study the pathology and pathogenesis of ALS, including invertebrates and vertebrates, such as yeast, flies, worms, zebrafish, mice, rats, guinea pigs, dogs, and non-human primates. Although each model displays different phenotypes, these genetic models are complementary to the pathological mechanisms of motor neuron (MNs) degeneration and the progression of ALS and are thus beneficial for research on the potential common pathogenesis of ALS through different genetic animal models, furthering the search for novel medicine treatments (Bonifacino et al., 2021).

Commonly used invertebrate genetic models mainly consist of yeast, *Drosophila*, and *Caenorhabditis*. These invertebrate experimental models were originally used to study genetics and development. These were also sometimes used to study the mechanisms associated with molecular and cell biology and are now presently propelled into the study of functional genomics and proteomics. The highly manipulable genomes of these models allow us to rapidly reproduce the transgenic lines, providing the ideal models for studying gene functions and gene and protein network interaction (Surguchov, 2021).

The full sequence of the invertebrate model genome is also easy to access for comparison with higher vertebrates and mammals in facilitating the evolution of genomic studies to rapidly produce transgenic animals by DNA transformation. Invertebrate organisms have more significant experimental advantages than their mammalian counterparts, such as a short generation time, small size, easy maintenance, and low cost. Another advantage of the invertebrate model is that it is amenable to genetic studies forward from phenotype to gene and reverse from gene to phenotype. Classic forward genetics studies of invertebrate models allow us to identify new molecules or pathways involved in special cell processes, which is a key advantage of invertebrate models. Forward genetics studies that apply a chemical mutagen to invertebrate models can generate target gene mutants to elucidate target gene functions. Reverse genetic studies using invertebrate models can rapidly identify possible molecular and biological pathways of certain target genes. The knockdown of target gene mutants using RNA interference (RNAi) technologies in invertebrate models can dramatically reduce target gene products by introducing double-stranded RNA (dsRNA) in models and provide related data about the roles of some target genes in biological processes.

**Figure 1:**
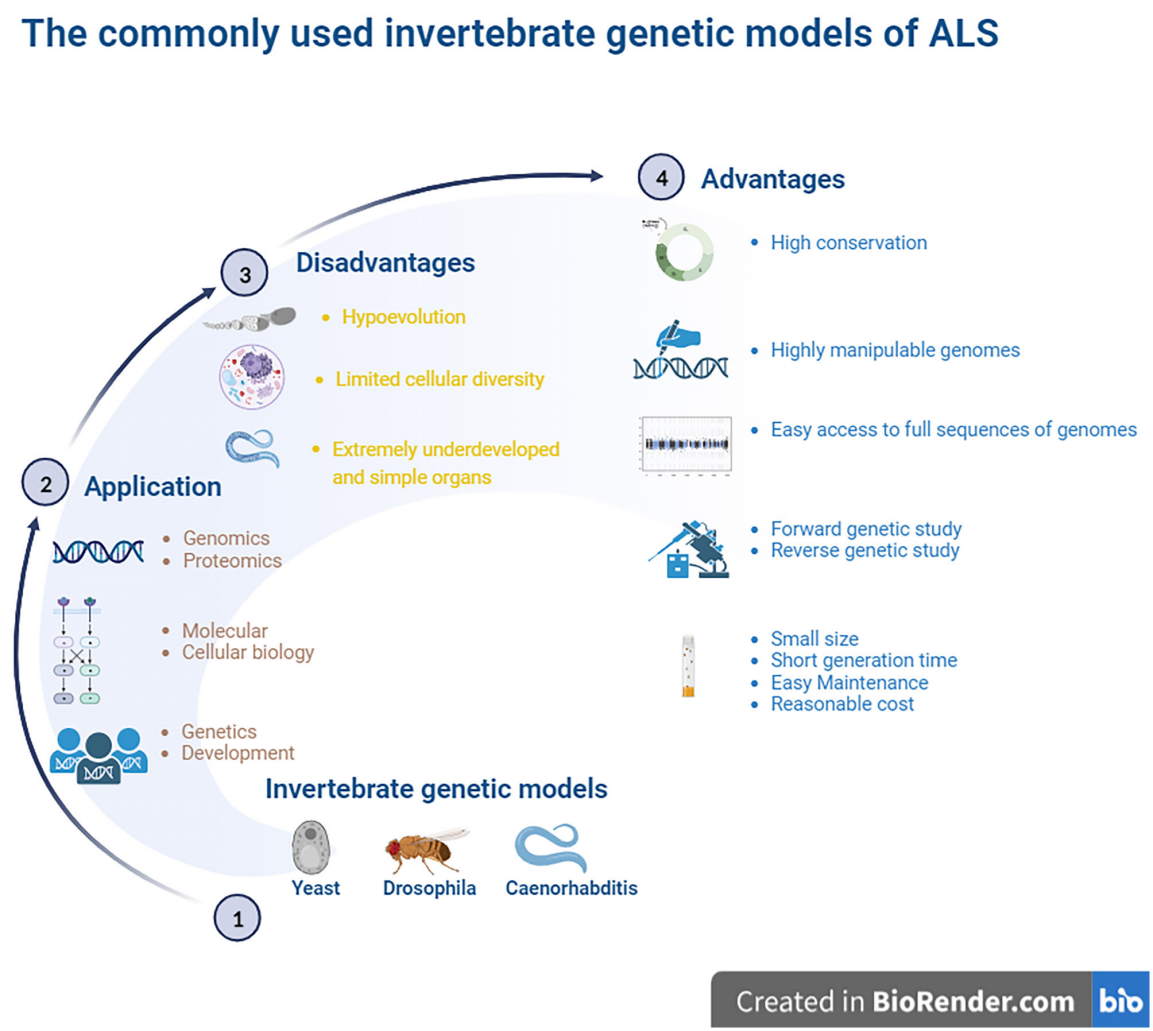
The commonly used invertebrate genetic models of ALS. The highly manipulative genomes of these models allow us to rapidly reproduce the transgenic lines for providing the ideal models for studying the gene functions and protein network interactions. The finds from the established models identified some key genes to involve in cell division, differentiation and apoptosis, which structures and functions were the high conservation with higher animals including humans, and it is easy access to the full sequence of model genomes. The model organisms have more significant experimental advantages, such as the short generate time, small sizes, easy maintenance as well as less cost. Besides, it is amenable to the genetic approaches of both forward and reverse. However, these invertebrate animal models are evolutionarily far from higher animal and mammalians, therefore, lots of physiologic functions can't be observed. The organs are extremely undeveloped as well as the limited cellular diversity, so it isn't beneficial to study the pathological alteration of organs and cellular levels. ALS, amyotrophic lateral sclerosis.

In addition to being amenable to forward and reverse genetic approaches, the advantages of invertebrate models include the following: yeast models possess a controlled environment, with a single system, an individual cell or tissue mechanism, easy gene manipulation, and easy culture. A special advantage of *Drosophila* is that it possesses large chromosomes, while a unique advantage of *Caenorhabditis* is its transparent body. Brenner (1974), Horvitz and Sulston (1980), and Sulston and Horvitz (1981) were awarded the Nobel Prize in Physiology or Medicine in 2002 for their seminal contributions concerning the genetic regulation of organ development and programmed cell death using the *Caenorhabditis elegans* (*C. elegans*) model as an investigative tool. The findings from these models established by them revealed some key genes involved in cell division, differentiation, and apoptosis and confirmed that both the structures and functions of these genes were highly conserved in higher animals, including humans. Based on their findings, they provided a framework in which simple animals can be used to identify potential molecular pathways and related biological processes. In addition, the advantages of *Drosophila* and *Caenorhabditis* also include a short life cycle, high fertility, a less complex nervous system, fully sequenced genome, which is homologous by more than 65%-75% to human genes, molecular pathways similar to humans, availability of mutagenic and transgenic techniques, and low cost (Gois et al., 2020). An important advantage of these models is that their data and information are often directly applied to research mechanisms of human diseases. Another advantage of establishing a model that imitates human disease using small invertebrate organisms is the high degree of conservation in mammalian organisms, which is useful for identifying the molecular components involved in pathogenesis. However, a major disadvantage of invertebrate models is that they are evolutionarily distant from mammals. In addition, compared to higher animals, the organs of invertebrate models are extremely undeveloped and simple, and alterations in many physiological functions and pathological and pathophysiological processes during the disease course in higher animals and mammals cannot be observed in invertebrate models. Another major disadvantage is the limited cellular diversity, which is not beneficial for studying pathological alterations at the organ and cellular levels (Gois et al., 2020) (Figure 1).

## 2 Genes involved in ALS

The majority of ALS cases are sporadic, and ~5%–10% of ALS cases are familial, with accurate Mendelian hereditary features and obvious penetrance (Gros-Louis et al., 2006). Since several missense mutations in the Cu^2+^/Zn^2+^ superoxide dismutase 1 (*SOD*1) gene were discovered in the subsets of familial ALS patients in 1993 (Rosen et al., 1993), various ALS-related gene mutations have been reported in the pathogenesis of ALS. Based on current genetic evidence, genetic mutations are thought to be the key cause of ALS pathogenesis. Mutations in some RNA metabolism-related genes and their related potential pathogenic mechanisms, such as disorders of cell transportation, axon outgrowth, protein metabolism, glutamatergic signaling, angiogenesis, neurotransmission, and antioxidant functions, have been confirmed to participate in the pathogenesis of ALS (Rosen et al., 1993; Neumann et al., 2006; Kwiatkowski et al., 2009; Renton et al., 2011; Morgan and Orrell, 2016). Mutations in *SOD1* (Rosen et al., 1993), TAR DNA-binding protein-43 (*TDP-43*), fused in sarcoma/translocated in liposarcoma (*FUS/TLS, FUS*) (Zou et al., 2017), and open reading frame 72 on chromosome 9 (*C9ORF72*) genes (DeJesus-Hernandez et al., 2011) are common in major cases of ALS. In addition, several genes with less frequent mutations, such as *VABP, OPTN, VCP, UBQLN2, MATR3, TBK1, NEK1, C21ORF2, ANXA11*, and *KIF5A* are also found to participate in the pathogenesis of partial ALS patients (Leblond et al., 2014; Chia et al., 2018; Brenner and Freischmidt, 2022). Mutations in ~30 genes have already been identified in patients with ALS to date; among them, major pathological mechanisms of the known ALS genes have been identified, including *SOD1, TDP43, FUS* and *C9orf72*. Recently discovered ALS-related candidates and risk genes include *SPTLC1, KANK1, CAV1, HTT*, and *WDR7* (Brenner and Freischmidt, 2022). To investigate the possible genetic mechanisms underlying ALS pathogenesis, various models have been established, including invertebrate models. In this review, we briefly discuss the usage, advantages, disadvantages, costs, and availability of current common invertebrate genetic models.

## 3 Nomenclature yeast models

Yeast models of ALS have been widely adopted to model human neurodegenerative diseases, such as ALS, since these have the advantages of easy manipulation and recapitulation compared to more complex eukaryotic cells. In particular, yeast models offer special insights into the potential relationships between the gain-of-function (GOF) toxicity of some protein aggregations. Although yeast models cannot reproduce the numerous pathogenic mechanisms involved in the degeneration of neuronal networks, they have proven to be ideal for discovering ALS-related pathogenic proteins. Among them, the yeast models of prion-like neurodegenerative disease-related proteins provide clues and valuable information for future research using a higher model system and ultimately developing therapeutics (Monahan et al., 2018).

**Figure 2:**
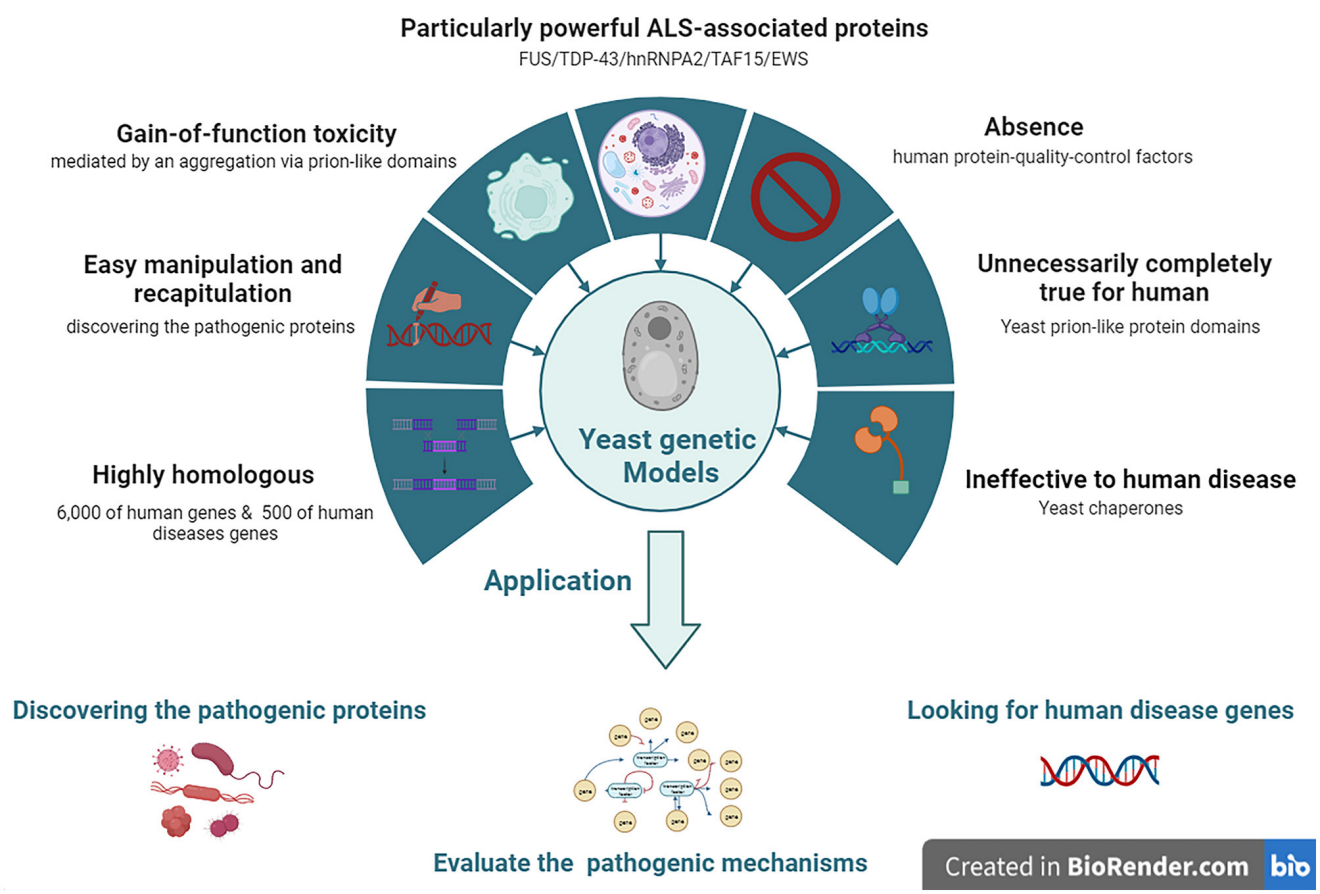
The features and applications of Yeasts genetic models in ALS. ALS, amyotrophic lateral sclerosis; EWS, Ewing sarcoma breakpoint region 1; FUS, fused in sarcoma; hnRNPA2, heterogeneous nuclear ribonucleoprotein A2; TAF15, TATA-box binding protein associated factor 15; TDP-43, TAR DNA-binding protein 43.

Approximately 6,000 human genes are homologous to those of yeast; among them, ~10% can be complemented with yeast (Cherry et al., 2012). Moreover, ~500 genes involved in human diseases are orthologs in yeast (Kryndushkin and Shewmaker, 2011). The most straightforward method for searching candidate pathogenic genes of human diseases among yeast is in situations where the disease genes are complemented by the yeast ortholog genes. The absence of human protein quality control factors in yeast can be an advantage in yeast models. Because of the deficiency of chaperones that regulate human pathogenic proteins among yeast, they can be useful for evaluating the chaperone-mediated changing mechanisms of aggregation and toxicity of human pathogenic proteins (De Graeve et al., 2013; Kumar et al., 2018; Park et al., 2018) (Figure 2).

### 3.1 Yeasts carrying *SOD1* and *OPTN* mutations

Many neurodegenerative disease-related genes/proteins such as *SOD1* and *OPTN* in ALS (Rabizadeh et al., 1995; Kryndushkin et al., 2012) have been established as models among yeast (Braun, 2015). To develop *SOD1* yeast models, various ALS-related *SOD*1 mutations such as *A4V, G37*R, *H48Q, G93A*, and *S134N* have been introduced into the yeast *SOD*1 gene. The *SOD1* yeast model demonstrated that the mutant human *SOD1* (*hmSOD1*) protein is unstable and reduces cell viability but does not form insoluble *SOD1* protein aggregates. Moreover, the *hmSOD1* toxic effects seem not to depend on mitochondrial dysfunction or oxidative stress, but rather on the inability to control central metabolic processes, which is most probably due to the severe disruption of vacuolar compartments (Bonifacino et al., 2021).

### 3.2 Yeasts carrying *FUS* and *TDP-43* mutations

ALS-related proteins with prion-like domains have proven to be particularly powerful in yeast models because they are similar to naturally existing yeast prion-like proteins, such as *FUS, TDP-43*, heterogeneous nuclear ribonucleoprotein A2 (*hnRNPA2*), TATA-box binding protein associated factor 15 (*TAF15*), and Ewing sarcoma breakpoint region 1 (*EWS*) proteins, which have similar protein architectures. Moreover, these proteins were found in the neuronal cytoplasm of post-mortem ALS patients. In yeast models, ALS-related proteins and their mutant isoforms typically induce GOF toxicity, which is partially mediated by aggregation via prion-like domains. Yeast did not possess *TDP-43* orthologous genes. Yeast models overexpressing human wild-type (WT) *TDP-43* showed that TDP-43 accumulated and formed subcellular aggregates in the cytoplasm, which inhibited cell growth, disrupted cell morphology, and generated cytotoxicity. Yeast models of ALS-related *TDP-43* mutations, such as *K, M337V, Q343R, N345K, R361S*, and *N390D*, accelerate protein aggregation, increase the number of cytosolic aggregates, and lead to growth arrest and cell death. Yeasts do not express *FUS* orthologs; thus, yeasts *FUS* models have been established by ectopically transforming human WT or mutant *FUS* genes into yeast. *FUS* yeast models are tightly associated with the translocation of *FUS* protein from the nucleus to the cytoplasm, forming aggregates co-localized with *P* bodies and stress granules in yeast cytoplasm and inhibiting the ubiquitin proteasome systems (Bonifacino et al., 2021).

Yeast models also have some limitations and disadvantages. Yeast protein domains are not the same as those in humans; thus, they must be carefully considered in studies using yeast models. The formation of prion-like proteins in yeast is its (True et al., 2004; Halfmann et al., 2012); however, this is not the case for human ALS proteins with prion-like domains. Almost all mammalian all mammalian prion-like proteins are harmful to humans (An and Harrison, 2016) since the properties of yeast prion-like protein domains are not completely conserved, but produce distinct alterations of constructs and functions that lead to contrary functions between yeast and humans during the human evolution process. Another limitation of yeast models is yeast cells do not have resident interacting proteins or chaperones that regulate human pathogenic proteins compared to mammalian cells. Chaperones are especially important for quality control and are integral to the propagation of endogenous prion proteins. Owing to the deficiency of resident interacting proteins and chaperones in yeast, the interaction of ectopically expressing human prion-like proteins in yeast is naturally different from that in humans; thus, the conclusions derived from studying protein interactions in yeast models may not be suitable for human diseases (Masison and Reidy, 2015).

## 4 *Drosophila melanogaster* models

**Figure 3:**
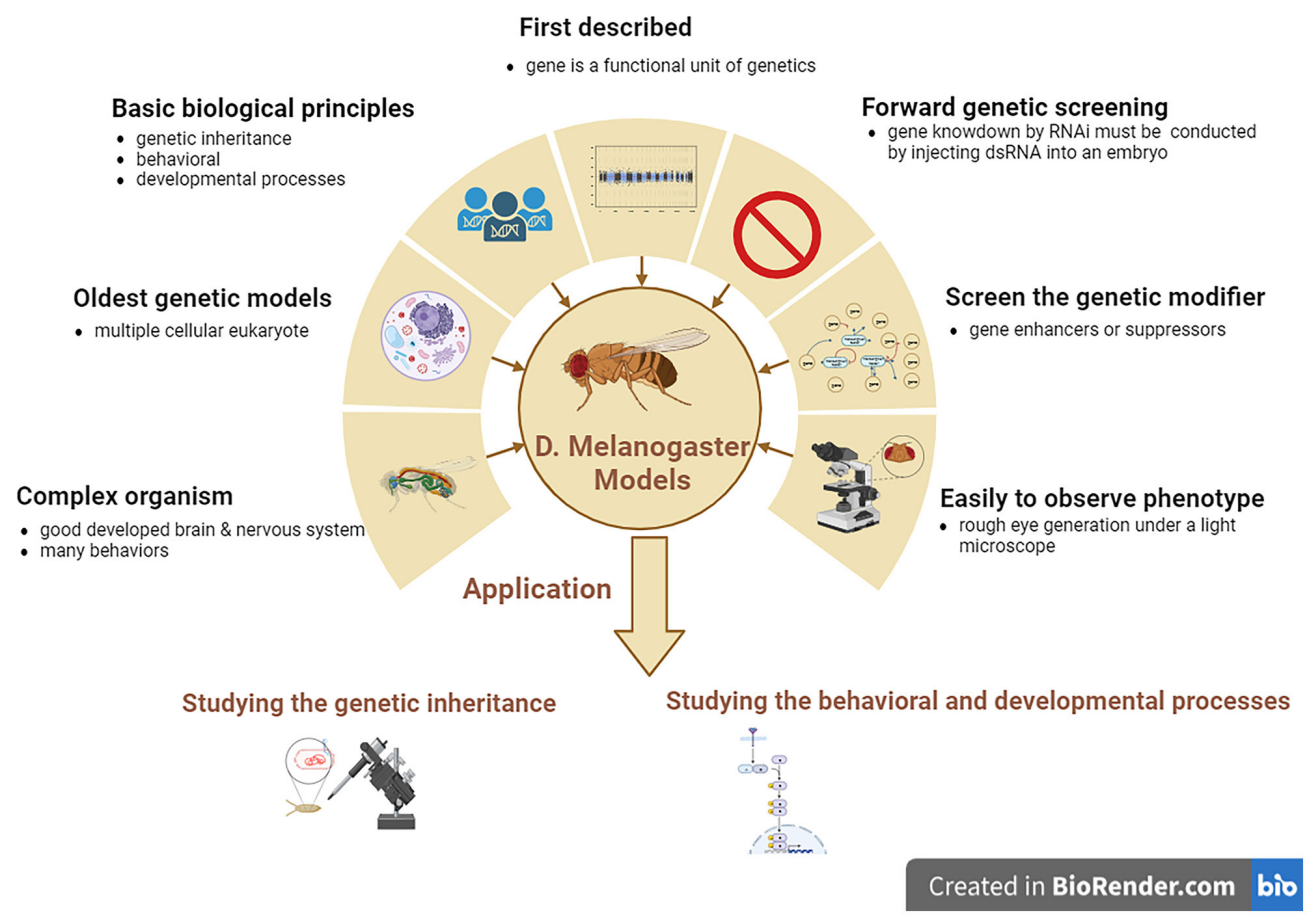
The features and applications of *D. melanogaster* genetic models in ALS. ALS, amyotrophic lateral sclerosis; *D. melanogaster, Drosophila melanogaster*.

*Drosophila melanogaster* is a good genetic model for studying neurodegenerative diseases, including ALS. *D. melanogaster* is a relatively complicated animal with a well-developed brain and other neural systems and display various behaviors such as learning, motor, and vision. *D. melanogaster* models are the earliest multiple cellular eukaryotes used as genetic models and have been applied in studying various basic biological principles, such as genetic phenotypes and genotypes, and behavioral and developed processes. The gene was first described as a functional genetic unit in *D. melanogaster* models. These models can be used to easily observe the neurodegenerative phenotype of rough eye generation under a light microscope. In addition, it can also be used to screen for genetic modifiers of gene enhancers and suppressors and can be used to study genetic inheritance and behavioral and developmental processes associated with human neurodegenerative diseases, including ALS. However, one main disadvantage of *D. melanogaster* models is that forward genetic screening by RNAi is relatively complex compared to the *C. elegans* model. Gene knockdown by RNAi cannot be conducted by feeding with dsRNA as simply as in *C. elegans* model; therefore, *D. melanogaster* models are made by injecting dsRNA into their embryos (Figure 3).

### 4.1 *D. melanogaster* carrying *SOD1* mutations

The *hSOD1 D. melanogaster* models were produced by amplifying the *hSOD1* gene through missense mutation technology and replacing the nitrogenous base among DNA correspondence for *hSOD1* reading site with a directive mutation. The *hmSOD1 D. melanogaster* models exhibit movement dysfunction, local accumulation of *hmSOD1* in MNs, enlargement of glial cells, neuronal degeneration, muscle contraction, decreased survival rates, and mitochondrial dysfunction (Gois et al., 2020). It reduces lifespan and fecundity and increases susceptibility to oxidative stress, movement defects, and necrotic cell death in *hmSOD1 D. melanogaster* models with *SOD1* mutations (Phillips et al., 1989). The *hmSOD1 D. melanogaster* model expresses four *hSOD1* mutants – *G85R, H71Y, H48R* and *G37R*. Among the endogenous *D. melanogaster, SOD1* (*dSOD1*) produces a large amount of *dSOD1* protein in neural cells (Layalle et al., 2021). Models expressing *G85R, H71Y*, and *H48R* mutations show decreased survival, developmental defects, and larval and adult dyskinesia due to muscle axon contraction (Braems et al., 2021). In addition, some antioxidant compounds have neuroprotective effects that improve exercise performance, prolong lifespan, and reduce *hSOD1* cytoplasmic inclusion bodies in the *hSOD1 D. melanogaster* model (Liguori et al., 2021).

The transgenic *D. melanogaster* model expressing only *hSOD1*^*WT*^ in MNs has been demonstrated to extend its longevity, but does not affect the movement or survival of MNs (Parkes et al., 1998). Moreover, the *hSOD1*^*WT*^transgenic *D. melanogaster* model extended the longevity of the *SOD1* deletion mutant and normal *D. melanogaster* but did not prevent age-dependent movement disorders. The *D. melanogaster* model's widespread downregulation of *SOD1* expression accelerates age-dependent movement disorders and shortens the lifespan of *D. melanogaster* (Braems et al., 2021). Meanwhile, the *D. melanogaster* model selectively expressing WT or causative*-*related *hSOD1* mutations (*A4*V and *G85R*) in MNs induces progressive movement dysfunction with electrophysiological defect, and the *SOD1* aberrant accumulates stress responses around the glial cells (Watson et al., 2008). Moreover, the transgenic *D. melanogaster* model specifically expressing *hmSOD1* in MNs showed a progressive motor protective effect, prevented the accumulation of *hmSOD1* aggregation, and increased the glial cell stress response in the ventral nervous cord, accompanied by electrophysiological defects in neural circuits. The *hmSOD1* transgenic *D. melanogaster* overexpressing mutated *SOD1* can model both cellular and non-cellular autonomic damage (Walters et al., 2019). The transgenic *D. melanogaster* model expressing WT or mutant (A4V and *G85R*) *hSOD1* among *D. melanogaster* MNs would lead to *D. melanogaster* climbing defects that progress over time and defects among neural circuits accompanied by the stress responses of glial cells and local accumulation of mutant SOD1 proteins among MNs (Braems et al., 2021). Meanwhile, the *hSOD1 A4V* transgenic *D. melanogaster* model showed synaptic transmission defects and focal mutation *SOD1* accumulation in MNs, HSP upregulation in glial cells, cellular autonomic lesions in MNs, and abnormal alterations in glial cells. This *D. melanogaster* model reveals that ALS is not confined to damaging MNs, and toxic *SOD1* transfers from neurons to glial cells (Clement et al., 2003; Boillée et al., 2006). The *hSOD1* transgenic *D. melanogaster* models are usually established by *hSOD1* gene expression among the MNs of *D. melanogaster SOD1* null background applying GAL4/UAS yeast systems. In *hSOD1* transgenic *D. melanogaster* models, very low levels of *hSOD1*^*WT*^ expression are sufficient to reverse the lifespan reduction, oxidative stress increase, and physiological dysfunctions related to the *SOD1* null *D. melanogaster* model (Mockett et al., 2003). In patients with ALS, it is very difficult to identify the complex genetics involved in its pathogenesis and to functionally investigate new candidate disease genes. *D. melanogaster* models can allow for an in-depth study of ALS progression from the earliest signs to terminal stages. They can rapidly perform a large range of simple assays from lifespan and motor assays to anatomical screens and can provide important functional information on the potential machinery required for proper motor neuron function and how this machinery may be dysregulated during ALS, which would not be possible in human neural systems. The *D. melanogaster* model is useful for addressing complex genetic diseases such as ALS. The UAS/Gal4/Gal80 system can upregulate and knock down *D. melanogaster* genes, including *SOD1*, and the ectopic expression of human *SOD1* genes or mutations in a tissue-specific manner, exhibiting the typical pathologies of *hmSOD1* ALS, which is beneficial for further investigation to identify disease-modifying genes, mutations, and disease pathways (Walters et al., 2019).

### 4.2 *D. melanogaster* carrying *TDP-43* mutations

TDP-43 is a highly conserved and ubiquitously expressed nuclear protein that participates in various cellular processes, including mRNA splicing, transcription, stability, and transportation (Langellotti et al., 2018; Lembke et al., 2019). TDP-43 is crucial for promoting the formation and growth of neuromuscular junctions (NMJ) (Strah et al., 2020). A number of *D. melanogaster* models generated by the TDP-43 toxicity of endogenous *D. melanogaster TDP-43* (*dTDP*) and *hTDP-43* transgenic expression revealed that the *TDP-43* protein showed toxicity, and the major phenotypes were largely similar in these *D. melanogaster* models. The *D. melanogaster* model lacking *dTDP* appears externally normal, but presents a deficiency in locomotion behaviors, a reduction in lifespan, anatomical defects of NMJ, and a decrease in dendrite branches (Feiguin et al., 2009; Lu et al., 2009; Lin et al., 2011). Among the above-mentioned *TDP-43 D. melanogaster* models, these phenotypes can be recovered and repaired by expressing the normal hTDP-43 protein in neurons, such as MNs (Feiguin et al., 2009). The phenotypes of *TDP-43 D. melanogaster* models revealed that the dysfunction of *TDP-43* might result in the pathogenesis of ALS. In addition, overexpression of either *dTDP* or *hTDP-43* in *D. melanogaster* also displayed the main pathological characteristics of ALS, premature lethality, neuronal loss, and defects in NMJ architecture and movement. *TDP-43 D. melanogaster* models revealed that the mechanisms of toxic GOF are related to *TDP-43* proteinopathy (Lu et al., 2009; Elden et al., 2010; Hanson et al., 2010; Li et al., 2010; Ritson et al., 2010; Voigt et al., 2010; Estes et al., 2011; Miguel et al., 2011).

To study and identify the possible roles and potential pathogenic mechanisms of the *TDP-43* protein in the pathogenesis of ALS, several *TDP-43* transgenic *D. melanogaster* models have been developed to further explore whether gene modification of *TDP-43* can increase or decrease the generation of *TDP-43* toxicity (Elden et al., 2010; Hanson et al., 2010; Ritson et al., 2010). In these *TDP-43* transgenic *D. melanogaster* models, upregulation of the human *ATXN2* gene ortholog Pab1-binding protein 1 enhanced *TDP-43* toxicity, resulting in more severe *TDP-43*-induced phenotypes (Elden et al., 2010). Moreover, the *TDP-43* interacting partner *ubiquilin 1* overexpression also produces similar *TDP-43*-induced phenotypes (Kim et al., 2009), reduced steady expression of *TDP-43*, and enhanced *TDP-43* phenotypes (Hanson et al., 2010). The *TDP-43*-induced phenotypes among *TDP-43* transgenic *D. melanogaster* models can also be modulated by co-expressing an ATPase member, valosin-containing protein (VCP), which is related to the protein family members of cellular activities that regulate many cell processes (Ritson et al., 2010). The above-described *TDP-43* transgenic *D. melanogaster* models may enable the development of novel therapeutic targets that regulate *TDP-43* expression in *TDP-43*-associated ALS patients, which is expected to prevent or treat *TDP-43*-associated ALS patients.

Bis-(2-ethylhexyl)-2,3,4,5-tetrabromophthalate (TBPH), a *D. melanogaster TDP-43* homolog, promotes target gene transcription by combining with sequences rich in uridine guanine, thus stabilizing the binding of chromatin regulators to DNA (Zhang et al., 2018a). Overexpression of mutant or WT *hTDP-43* and TBPH in transgenic *D. melanogaster* models affects lifespan, mobility, axonal transport, and pupal shell sealing, whereas TBPH depletion leads to movement disorders and a shortened lifespan (Walters et al., 2019; Liguori et al., 2021). Among them, *hTDP-43*^*WT*^ overexpression in neurons results in an increase in NMJ buttons and branches associated with hTDP-43^WT^ protein aggregation (Kankel et al., 2020). However, the phenotypes induced by the mutant *hTDP-43* were not distinguishable from the *hTDP-43*^*WT*^ induced phenotypes (Feldman et al., 2022).

Increased *TDP-43* expression in *TDP-43* transgenic *D. melanogaster* models induces mitochondrial dysfunction, including ridge abnormality and loss, resulting in a decrease in mitochondrial membrane potential and an increase in reactive oxygen species (ROS) production, usually accompanied by the loss of striated muscle tissue, and affects the survival and function of neurons due to excessive ROS production. Moreover, the *TDP-43* expression increase also inhibited mitochondrial complex I activation and reduced mitochondrial ATP synthesis. Compared with the *D. melanogaster* control model, the volume of mitochondria in the eyes of *D. melanogaster* expressing *TDP-43*^*WT*^ or *TDP*−43^*A*315^T was significantly reduced. *TDP-43*^*WT*^ and *TDP-43*^*A*315*TGOF*^ induced mitochondrial unfolded protein responses, including Lon and LonP1. LonP1 is a major mitochondrial matrix protease that interacts with *TDP-43* to reduce mitochondrial *TDP-43* protein levels, which can prevent mitochondrial damage and neurodegeneration in the *D. melanogaster* model induced by *TDP-43* (Khalil et al., 2017; Wang et al., 2019; Layalle et al., 2021). Highly fragmented mitochondria have also been found among the axons of MNs in a *D. melanogaster* model expressing *TDP-43* (Layalle et al., 2021). Sigma-1 receptor enhances the resistance of *D. melanogaster* to oxidative stress and exerts positive effects on motor activity and ATP levels (Couly et al., 2020).

In *TDP-43 D. melanogaster* models, significant changes were observed in glucose metabolism, including an increase in pyruvic acid levels. Among the MNs of *TDP-43 D. melanogaster models*, the mRNA level of phosphofructose kinase (an enzyme that controls the rate of fermentation) significantly increased (Manzo et al., 2019; Layalle et al., 2021). A high sugar diet can improve the exercise and life defect induced by *TDP-43* protein lesions among MNs or/and glial cells, but it does not improve the muscles lesions in the *TDP-43 D. melanogaster* models, indicating that the metabolic disorders participate in the neural systems damage (Manzo et al., 2019). In addition, it has been revealed that some changes in the lipid metabolism, such as the reduction of both carnitine shuttle and lipid β oxidation, occur among *TDP-43 D. melanogaster* models (Layalle et al., 2021).

Among *TDP-43* transgenic *D. melanogaster* models, other factors were found to have an impact on *TDP-43*. For example, the inhibition of protein tyrosine kinase 2 (PTK2) significantly reduces ubiquitin aggregation and cytotoxicity of *TDP-43*. Non-phosphorylated sequencesome 1 (SQSTM1) inhibits the accumulation and neurotoxicity of insoluble polyubiquitin proteins induced by *TDP-43* overexpression in neuronal cells. TANK-binding kinase 1 (TBK1) participates in PTK2-mediated SQSTM1 phosphorylation. Therefore, the PTK2-TBK1-SQSTM1 axis plays a key role in the pathogenesis of *TDP-43* by modulating neurotoxicity caused by damage to the ubiquitin-proteasome system in the *TDP-43* transgenic *D. melanogaster* model (Lee et al., 2020). Recently, it was found that the generation of reversible droplet-like nuclear bodies (Wang et al., 2020), the overexpression of long non-coding RNA nuclear enriched abundant transcript 1–1 (Matsukawa et al., 2021), *Mucuna pruriens* and *Withania somnifera* (Maccioni et al., 2018; Paul et al., 2021; Zahra et al., 2022), and the production of *CG5445* (a previously uncharacterized *D. melanogaster* gene) (Uechi et al., 2018, p. 3) alleviate the cytotoxicity of *TDP-43* in neurons and improve movement or eye symptoms in the *TDP-43* transgenic *D. melanogaster* model.

Gemin3 (DDX20 or DP103) is a dead-box RNA helicase that participates in a variety of cellular processes. The combination of Gemin3 and *TDP-43* or *FUS* destruction aggravates vitality defects, motor dysfunction, and muscle atrophy while inhibiting the overgrowth of NMJ in the *TDP-43* transgenic *D. melanogaster* model (Cacciottolo et al., 2019). A 3-fold knockdown of the lethal gene inhibitor (elongation factor of RNA polymerase II) significantly inhibited the morphological defects of the compound eye and medial retinae induced by *TDP-43* in *TDP-43* transgenic *D. melanogaster* models. *D. melanogaster* has five histone deacetylases (HDAC), among which *Rpd3* knockdown significantly inhibits the rough eye phenotype caused by *hTDP-43* in the *TDP-43* transgenic *D. melanogaster* model (Yamaguchi et al., 2021). TBPH deficiency leads to larval death and reduced HDAC6 levels (Braems et al., 2021). Among the many single or double hydrophilic tag conjugating peptides D1-D8, D4 has the strongest ability to degrade *TDP-43* protein and can penetrate the cell wall in short periods, inducing *TDP-43* protein degradation in a dose- and time-dependent manner (Gao et al., 2019).

Reverse transposable element (RTE) expression was strongly upregulated in the head of TBPH-null *D. melanogaster*. TBPH regulates the silencing mechanism of small interfering RNA inhibiting RTE, and TBPH modulates Dicer 2 expression levels through direct protein mRNA interaction in *TDP-43* transgenic *D. melanogaster* models (Romano et al., 2020). TBPH also interacted with VCP to inhibit VCP-induced degeneration in a *TDP-43* transgenic *D. melanogaster* model (Braems et al., 2021).

### 4.3 *D. melanogaster* carrying *FUS* mutations

Although *FUS* mutations obviously affect MNs as well as other neurons found in *FUS* transgenic *D. melanogaster* models, its pathogenic mechanism of proteinopathy remains largely unknown. To study the alterations of *FUS*-associated functions in neurodegenerative diseases, including ALS, we reproduced the *FUS* transgenic *D. melanogaster* model expressing mutant human *FUS* (*hmFUS*), such as *R518K, R521C*, and *R521H*. These models cause a severe neurodegeneration in *D. melanogaster* eyes, whereas WT human *FUS* (*hFUS*^*W*^T) expression only led to mild neurodegeneration (Lanson et al., 2011).

Both movement defects and premature death have been observed in mutant *FUS* transgenic *D. melanogaster*. Moreover, mutant *FUS* overexpression increased the accumulation of the *FUS* protein. Causative roles on ALS-related *hFUS* mutants including both *R524S* and *P525L* in the *FUS* transgenic *D. melanogaste*r model have also been described (Chen et al., 2011). Among the *D. melanogaste*r models overexpressing WT and ALS-related *FUS* mutations among various neuron subsets, such as photoreceptors, mushroom bodies, and MNs, progressive age-related neuronal degeneration, such as axon loss, morphological alterations, and functional impairment of MNs, has been observed. The model of *Cabeza* (*hFUS D. melanogaster* homolog)-deficient *D. melanogaster* is used to study *FUS* functions, and the results showed both reduced lifespan and locomotor defects compared to controls. It was also found that transferring *hFUS*^*WT*^ gene into this model could fully recover these phenotypes, but in this model co-expressing the ALS-related mutant *FUS* proteins, the phenotypes of reduced lifespan and locomotor defects were not recovered. This suggests that ALS-linked *FUS* mutations are toxic in *FUS* transgenic *D. melanogaster* models (Walker et al., 2011). In a transgenic *D. melanogaster* model coupled *FUS* with *TDP-43* gene in neurons, it was demonstrated that *FUS* acts on ALS pathogen-associated effects together with TDP-43 via a common genetic pathway. Moreover, *FUS* and *TDP-43* exert synergistic effects on RNA-dependent complexes. These models show that *FUS* and *TDP-43* exert partial damage during the pathogenesis of ALS (Layalle et al., 2021).

*D. melanogaster FUS* homolog *Cabeza* is extensively expressed in the majority of tissues, including the neural system. Most *Cabeza*-*D. melanogaster* models with Cabeza functional loss are fatal, and only a few *D. melanogaster* are able to develop into adulthood, but exhibit severely shortened lifespans and motor disorders. Importantly, co-expression of wild-type *Cabeza* in the neurons of *Cabeza*-mutant *D. melanogaster* models can repair climbing and flight defects. Compared with *Cabeza*-mutant *D. melanogaster* models, larvae overexpressing *Cabeza*/*FUS*^*WT*^ exhibited opposite NMJ electrophysiological phenotypes, characterized by reduced excitatory junction potentials and miniature excitatory junction potential amplitudes. In addition, it was found that the *D. Melanogaster* model may have a self-inhibitory mechanism for *FUS/Cabeza*, as the overexpression of *FUS* reduces the intracellular *Cabeza* content (Zhang et al., 2018a). *D. melanogaster* overexpressing *hFUS*^*W*^T exhibits abnormal eye morphology, shortened lifespan, eclosion defects, climbing defects, a reduced number of synaptic buttons, a reduced active region of the NMJ, and axonal degeneration (Azuma et al., 2018). *hFUS* is overexpressed in photoreceptor neurons in the eyes of *D. melanogaster*, resulting in mild retinal degeneration, rough eye surfaces, and a decrease in red pigment. The changes in the different *FUS* regions have different effects, such as in HEK293 cells, where the C-terminal truncated *FUS* chelates the *FUS*^*WT*^ protein from the nucleus to the cytoplasm, thereby exacerbating *FUS*-induced retinal degeneration. The *FUS*-*P525L* mutation exacerbates *FUS*-induced retinal degeneration by increasing the *FUS* cytoplasmic distribution (Matsumoto et al., 2018; Layalle et al., 2021).

The arginine residue in the low-complexity domain of the *FUS* C-terminus is necessary for the maturation of *FUS*, while the N-terminal glutamine-glycine-serine-tyrosine (QGSY)-rich region (amino acids 1–164) and the C-terminal RGG2 domain are necessary for the toxicity of *FUS*, including the N-terminal QGSY, prion-like domains, and C-terminal RGG2 domains. It can distinguish between *FUS* and Cabeza toxicity based on its QGSY domain (Bogaert et al., 2018). The expression of different *FUS* mutations (*R521C, 521H, 518*K, *R524S*, or *P525*L) resulted in more severe rough-eye phenotypes in *FUS* transgenic *D. Melanogaster* models. *FUS* expression triggers Hippo activation and c-Jun N-terminal kinase signaling, leading to neuronal degeneration in *FUS* transgenic *D. Melanogaster* models. In addition, the Hippo signaling pathway is a modifier of *Cabeza* knockdown phenotypes (Layalle et al., 2021).

In *FUS* transgenic *D. melanogaster* models, it has been shown that inhibiting nuclear output can reduce *hFUS*-induced toxicity, thereby confirming the involvement of nuclear-cytoplasmic transport in the pathogenesis of ALS. It is worth noting that, recently, a series of complex enhancers and inhibitors have been found for ALS modifying factors screened in two different transgenic *D. melanogaster* models carrying human mutant *FUS* genes (Liguori et al., 2021). In the *Cabeza* knockdown *D. melanogaster* models, there are 14 inhibitory mutations knockdown models that effectively inhibit rough eye phenotypes caused by *Cabeza* knockdown. Mutations in Chameau and N-alpha-acetyltransferase 60 inhibit locomotion and cause morphological defects in NMJ synapses caused by *Cabeza* knockdown (Yamaguchi et al., 2021). *FUS* is similar to *TDP-43* gene and regulates synaptic transmission among *D. melanogaster* NMJ; synaptic transmission defects precede the degeneration and loss of MNs in *FUS* transgenic *D. melanogaster* models. The nuclear-cytoplasmic transport proteins exportin-1 and nucleoporin-154 are modifiers of FUS toxicity, which prevent FUS-induced toxicity and reduce apoptosis of ventral nerve cord neurons (Braems et al., 2021).

In third-instar larvae of a *Cabeza* mutant *D. melanogaster* model, it was shown that the *Cabeza* mutant leads to an increase in induced and spontaneous neurotransmitter release in the NMJ (Zhang et al., 2018a). In addition, overexpression of *hFUS* led to a decrease in presynaptic activity areas and impaired synaptic transmission in *hFUS* transgenic *D. melanogaster* model. The loss and acquisition of *FUS* function in *FUS* and *Cabeza* transgenic *D. melanogaster* models may be achieved through dominantly negative mechanisms or the downregulation of endogenous *Cabez*a, which are mediated by interference with vesicular and mitochondrial transportation. This indicates that the *Cabeza* mutation leads to developmental and motor defects, which become more severe with age (Walters et al., 2019). Mutant *FUS* or *D. melanogaster* homolog *Cabeza* expressing type IV dendritic neurons leads to *FUS* or *Cabeza* cytoplasm mislocalization and axon transportation toward the pre-synapse end in *FUS* or *Cabeza* transgenic *D. melanogaster* models. Moreover, *FUS* or *Cabeza* overexpression leads to the progressive loss of neuronal projections and the reduction of synaptic mitochondria, while a large number of calcium transients appear in synapses by controlling the expression of calcium channels. In addition, mutant *FUS* overexpression results in a decrease in presynaptic synaptic-binding proteins, specifically disrupting axonal transport and inducing excessive excitability. Therefore, the mutation *FUS/Cabeza* damages the axon transportation of synapse vesicular proteins, thereby reducing the levels of synaptic-binding proteins, which are the main components of presynaptic release mechanisms. The overexpression of *FUS* can disrupt mitochondrial transport in *D. melanogaster* MNs, and an increase in FUS transport during neural biological processes alters the focal levels of synapse transcription or induces the formation of stress particles, thereby disrupting local translation and causing synaptic hyperexcitability (Machamer et al., 2018).

Karyopherin beta-2, also known as Kap-β-2 or transportin-1, participates in proline/tyrosine nuclear localization signal (PY-NLS), which inhibits and reverses the FUS fibrosis. In addition, it is worth noting that Kap-β2 can prevent RNA binding protein (RBP) with PY-NLS from accumulating among stress granules, restore the nucleus RBP localization and function, and rescue the neurodegeneration induced by ALS-related FUS in the *FUS* transgenic *D. melanogaster* models. The increase of Kap-β2 saves neurodegeneration and lifespan related to *FUS* in the *FUS* transgenic *D. melanogaster* models and reduces the accumulation of *FUS* in stress granules (Guo et al., 2018).

Knockdown of heat shock response gense in the *D. melanogaster* model expressing *hFUS*^*WT*^ ω-LncRNA can reduce the level of *hFUS*^*WT*^ mRNA and induce the generation of insoluble inclusion bodies comprising non-toxic *hFUS*^*WT*^ proteins and lysosomal-associated membrane protein 1 in cytoplasm (Yamaguchi et al., 2021). Nuclear chromatin-binding protein (Xrp1), the main modifier of *Cabeza* mutative phenotypes, is strongly upregulated in the *Cabeza*-mutant *D. melanogaster* model, effectively reducing motor and life defects. Moreover, the *Cabeza*-mutant *D. melanogaster* model exhibits an imbalance in gene expression, and the Xrp1 heterozygosity can alleviate this (Mallik et al., 2018). Several genes, including *TDP-43, Transportin-1, TER94/VCP*, arginine methyltransferase, and the mitochondrial companion *Hsp60* have been shown to interact with *FUS/Cabeza* genetically in the *FUS/Cabeza* transgenic *D. melanogaster* model (Zhang et al., 2018a).

### 4.4 *D. melanogaster* carrying *C9orf72* mutations

*C9orf72* is a common pathogenic gene involved in ALS. The present study showed that different cellular processes, including transcription, translation, nuclear-cytoplasmic transport, and protein degradation, are involved in the pathogenesis of *C9orf72* ALS (Liguori et al., 2021). The *D. melanogaster* genome does not contain a direct homolog of human *C9orf72*, thus, an ALS-linked *D. melanogaster* model related to *C9orf72* was developed by expressing the GGGGCC (G4C2) repeat (Yamaguchi et al., 2021). Although *C9orf72* in ALS patients usually comprises over hundreds of G4C2 repeat sequences, the overexpression of 30–58 G4C2 repeat sequences in *D. melanogaster* eyes or MNs is sufficient to result in neuronal degeneration. Based on the expression level and time, the G4C2 repeat or arginine-rich dipeptide repeat (DPR) expressed among MNs leads to serious NMJ defects in the third-instar larvae of the *C9orf72* G4C2 repeat transgenic *D. melanogaster* model (Zhang et al., 2018a). The amplification of the G4C2 hexanucleotide repeat expansion (HRE) of *C9orf72* leads to the loss of *C9orf72* function by inhibiting transcriptional extension and splicing of the first intron (Zhang et al., 2018a). The characteristic pathological hallmark observed among the different tissues of *C9orf72* ALS, including MNs, is RNA lesions. RNA lesions are the result of HRE transcription, leading to the accumulation of repetitive RNA aggregates, usually located in the nucleus or cytosol (Layalle et al., 2021). This suggests that RNA-carrying HRE do not cause toxicity, but that the arginine-rich DPR sequence encoded by the HRE sequence mediates neurotoxicity (Moens et al., 2018). Consistent with this, the abnormal translation of HRE leads to the accumulation of DPR protein in the brains of ALS patients (Azoulay-Ginsburg et al., 2021). When comparing the effects of RNA expression among the HRE transgenic *D. melanogaster* model, only DPR proteins containing arginine have neurotoxicity on the G4C2 motifs encoding different dipeptide combinations (Bolus et al., 2020).

Poly (glycine-arginine) (GR) is a common genetic cause of ALS and FTD, occupying forty percent of fALS cases (Cunningham et al., 2020). Partial functional loss of an essential DNA repair protein Ku80 inhibits retinal degeneration induced by poly (GR) in poly (GR) transgenic *D. melanogaster* models. In the neurons of *D. melanogaster* model expressing poly (GR) and in patients with *C9orf72*, the Ku80 expression is significantly increased. Elevated Ku80 expression contributes to GR aggregation and induces neurodegeneration in a poly (GR) transgenic *D. melanogaster* model (Lopez-Gonzalez et al., 2019).

Arginine-rich DPR is a highly toxic product derived from the *C9orf72* repeat amplification mutation and is a common cause of fALS. However, their role in synaptic regulation and excitatory toxicity remains unclear. Applying *C9orf72* DPRs with different toxic intensities revealed that arginine-rich DPRs induced selective degeneration of glutamate neurons in the *C9orf72*-DPR transgenic *D. melanogaster* model. Among them, the *C9orf72*-DPR can stimulate synaptic overgrowth and promote glutamate release. Furthermore, an increase in glutamate release from excitatory neurons expressing DPR not only causes excitotoxicity in postsynaptic neurons, including MNs, but also produces cell-autonomous excitotoxicity in presynaptic neurons (Xu and Xu, 2018).

Up-frameshift 1 (UPF1) is an RBP with helicase activity that reduces DPR-mediated toxicity. Overexpression of UPF1 significantly reduces the severity of known neurodegenerative phenotypes and poly (GR) content without changing the amount of repeat RNA, indicating that UPF1 has a neuroprotective effect on *C9orf72*-ALS (Zaepfel et al., 2021). The present study shows that UPF1 expression regulates the DPR- or HRE-induced neurodegenerative phenotypes of *C9orf72*-ALS transgenic *D. melanogaster* models, including eye symptoms (Xu et al., 2019; Ortega et al., 2020; Sun et al., 2020). In addition, DPR can directly bind to nucleoporins, and knockdown of karyopherin alpha3 in the *C9orf72*-ALS transgenic *D. melanogaster* model can enhance the toxicity of DPR (Walters et al., 2019; Braems et al., 2021). Furthermore, the loss of C9orf72 function disrupts autophagy and lysosomal functions in many types of cells, including MNs (Ji et al., 2017; Shi et al., 2018; Zhu et al., 2020).

The key regulatory factor of autophagy, which is refractory to Sigma P/sequestosome 1 [Ref(2)P/p62], is an effective inhibitor of ALS induced by G4C2 HRE in *C9orf72* (Cunningham et al., 2020). The microphthalmia/transcription factor E family of transcription factors is a key regulator of autophagy-lysosome function. Transcription factor EB (TFEB) is a candidate therapeutic target for ALS (Cortes and La Spada, 2019). It was found that *C9orf72*-HRE impaired the microphthalmia-associated transcription factor/transcription factor EB nuclear input, disrupted autophagy, and exacerbated the protein balance defect in the *C9orf72* ALS transgenic *D. melanogaster* model. Increased TFEB activity may prevent neuronal death and promote recovery of neuronal function through different mechanisms in the *C9orf72*-ALS transgenic *D. melanogaster* model (Torra et al., 2018).

The G4C2 amplification repeat sequence and tau protein are traditionally considered to be related to partial clinical manifestations in several neurodegenerative diseases, such as ALS. Co-expression of the G4C2 repeat sequence and Tau can lead to the synergistic deterioration of rough eyes, movement functions, longevity, and abnormal NMJ morphology in the G4C2 repeat sequence and Tau co-expression transgenic *D. melanogaster* model. In addition, 30 G4C2 repeats increase tau phosphorylation levels (He et al., 2019). Moreover, the downregulation of *D. melanogaster* tau homologous protein can reduce neurodegeneration and motor lesions and prolong the lifespan of *C9ORF72* G4C2 repeatedly expressing *D. melanogaster* model (Braems et al., 2021).

## 5 *C. elegans* models

The *C. elegans* genome was the first multicellular organism to be completely sequenced in 1998 (*C. elegans* Sequencing Consortium, 1998). *C. elegans* contains ~20,000 genes that are highly similar to the 25,000 human genes. Therefore, it is considered as an ideal model for analyzing the genetic and molecular mechanisms underlying neuronal development, function, and disease. Moreover, more than 42% of human disease-linked genes have an organology with *C. elegans*, suggesting that almost all biochemical pathways have been conserved throughout the evolution of *C. elegans* and humans (Culetto and Sattelle, 2000). *C. elegans* was the first model used to study the biochemical pathways of human diseases.

*C. elegans* is genetically tractable, which indicates that its genome can be easily manipulated. This characteristic is particularly advantageous in ALS research, in which the identification of genetic factors contributing to the disease is essential. Researchers can introduce specific mutations associated with ALS into the *C. elegans* genome to observe the resulting phenotypic changes. This approach helps elucidate the genetic basis of ALS and identify potential therapeutic targets (Clarke et al., 2018). Twenty-six neurons in five different neuronal types are gamma-aminobutyric acid (GABA)-ergic neurons in *C. elegans* (McIntire et al., 1993). Among them, 19 GABAergic neurons, called type D neurons, exert regular locomotion by providing dorsal-ventral cross inhibition to the body wall muscle (White et al., 1986; McIntire et al., 1993). In addition, *C. elegans* contains more than 50 cholinergic MNs that control locomotion (Liu et al., 2020). Therefore, *C. elegans* transgenic models are useful for studying the pathological alterations related to locomotor dysfunction. *C. elegans* is a hermaphrodite, ~1 mm in length, with almost 3 days of short generation cycles, and ~300 progenies in a large brood. It has a short lifespan of ~3 weeks and undergoes rapid generation cycles, allowing for quick observation of the effects of genetic manipulations across multiple generations. In ALS studies, this characteristic enables researchers to assess the progression of neurodegeneration and the inheritance patterns of genetic mutations associated with the disease in a relatively short timeframe (von Mikecz, 2022). *C. elegans* has a transparent anatomical body that can be used to visualize all cell types at developmental stages (Brenner, 1974). The transparent body of *C. elegans* is another advantage, especially for the visualization of cellular and subcellular events. Researchers can observe the development and degeneration of neurons in real-time, providing valuable insights into the mechanisms of ALS pathogenesis. This transparency aids in monitoring changes, such as protein aggregation, cellular death, and alterations in neuronal morphology. The experimental techniques available for *C. elegans* make it easy to observe and quantify cellular events such as neuronal cell death and protein inclusion. This is critical for ALS research, in which an understanding of the dynamics of these events is essential. The *C. elegans* transparent body, combined with advanced imaging techniques, facilitates detailed analysis of cellular changes associated with ALS. Another advantage of the *C. elegans* model is the ease of observation and quantification of neuronal cell death and protein inclusion using experimental techniques (Ma et al., 2022).

The whole embryo cellular lineages of *C. elegans* are completely clear, which makes it possible to track organogenesis from the earliest stage of embryo formation to the terminal stage of organ differentiation and morphogenesis (Sulston et al., 1983). In addition, the nervous system of *C. elegans* is very simple; it possess 302 neurons among 959 cells in an adult *C. elegans* body. Each neuron is located in a unique anatomical position (Sulston et al., 1983). Twenty neurons are located in the larynx, while the 282 remaining neurons are located in various ganglia, from head to tail, along the main longitudinal axon tract in the ventral spinal cord. Among the neurons that develop during embryogenesis, 80 MNs develop at the post-embryonic stage. *C. elegans* has a relatively simple nervous system with 302 neurons, making it an excellent model for studying neural circuits and functions (Takeishi et al., 2020). Understanding the molecular and cellular events that underlie neurodegeneration is crucial for ALS research. The simplicity of the *C. elegans* nervous system allows researchers to map and monitor individual neurons, thereby facilitating the identification of key genes and pathways involved in ALS pathology (Gois et al., 2020).

The structures of the *C. elegans* nervous system were highly reproducible in each generation, as described previously, and high-resolution images were obtained by electron microscopic reconstruction. Approximately 5,000 chemical synapses, 2,000 neuromuscular junctions, and 500 gap junctions have been described, and all connections of the entire neuronal circuit have been mapped in *C. elegans* models (White et al., 1986). In *C. elegans* models, it is relatively easy to achieve target gene knockdown or overexpression by RNAi by injecting dsRNA into the unique genes of interest, simply soaking *C. elegans* in the dsRNA medium or feeding *C. elegans* the bacterium carrying the targeted dsRNA (Fire et al., 1998; Maeda et al., 2001). In addition, primary culture resources for neurons and muscle cells can also be obtained by dissecting *C. elegans*, which can further optimize and stabilize the growth of embryonic cells (Christensen et al., 2002).

**Figure 4:**
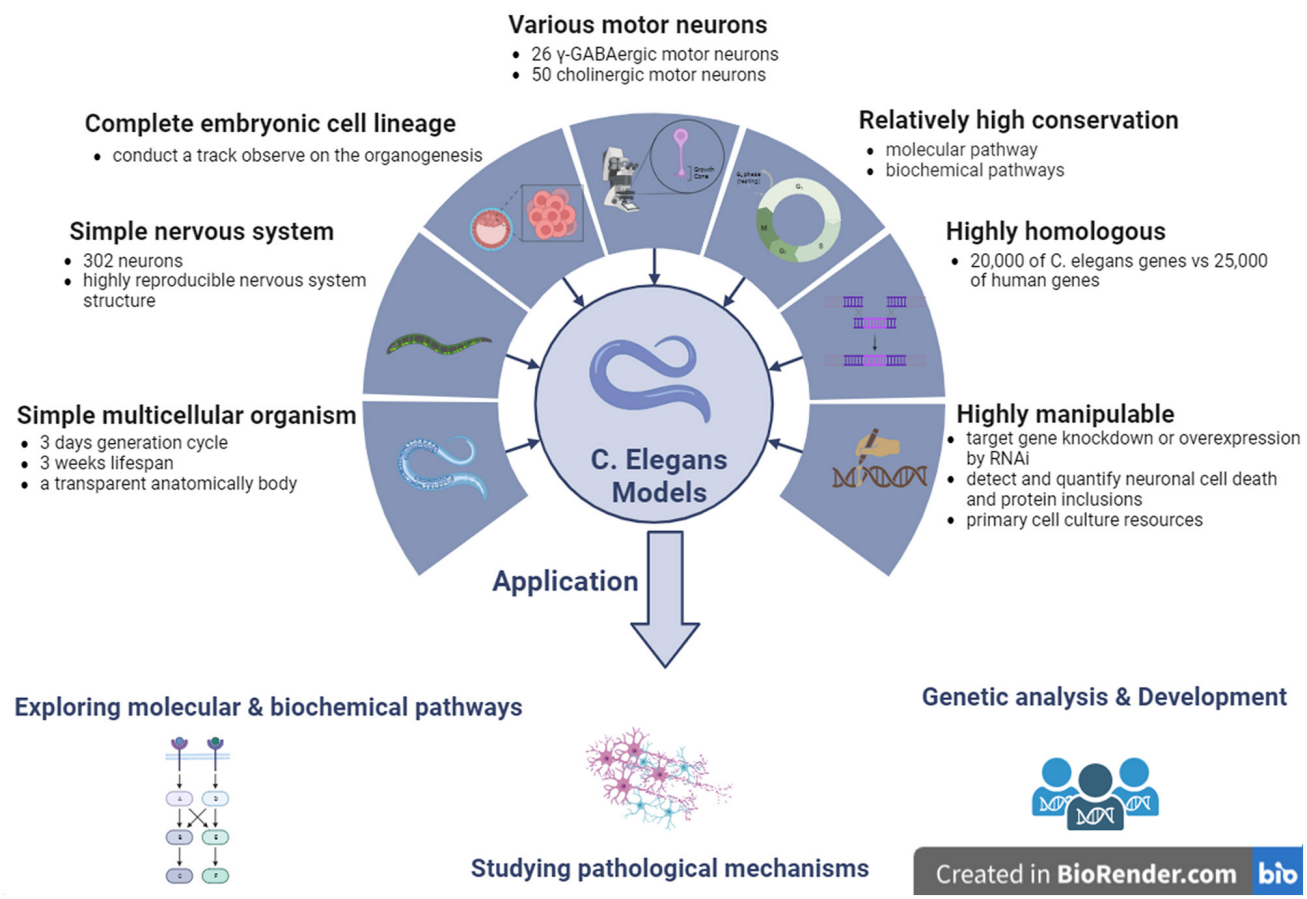
The features and applications of *C. elegans* genetic models in ALS. ALS, amyotrophic lateral sclerosis; *C. elegans, Caenorhabditis elegans*; γ-GABA, gamma-aminobutyric acid.

Although many disease-relevant genes have been identified in neurodegenerative disorders, including ALS, the mechanisms underlying the dysfunction and death of selective MNs remain poorly understood. Based on this, molecular conservation in the neuronal signaling pathway between invertebrates and vertebrates is high, and *C. elegans* contains almost all known signaling and neurotransmitter systems found in the nervous system of mammals (Bargmann, 1998). Therefore, *C. elegans* models have generally been used by researchers to investigate the mechanisms underlying neurodegenerative diseases, including ALS. Previous studies on *C. elegans* have provided valuable data and evidence for elucidating the molecular pathways involved in many human diseases, including ALS. Because of these advantages, *C. elegans* has attracted many researchers to use it as an *in vivo* model for researching pathological mechanisms in neurodegenerative diseases such as ALS, providing some perspective evidence for identifying potential pathogenic targets and developing new therapeutic measures against human diseases such as ALS (Figure 4).

### 5.1 *C. elegans* carrying *SOD1* mutations

Since *C. elegans* ALS models were first established in 2001 (Oeda et al., 2001), a series of *C. elegans* models have been developed, and many potential mechanisms associated with ALS pathogenesis have been identified. Among them, the *hmSOD1 C. elegans* model was produced by transferring wild-type human *SOD1* (WT*hSOD1*) and familial ALS (fALS) *SOD1* mutations, such as A4V, G37R, and G93A, by controlling several promoters of the heat shock protein (*HSP*)-16.2 and myosin heavy chain 3 (*myo-3*) genes. The *hmSOD1* transgenic *C. elegans* model controlled by the inducible HSP-16.2 promoter expresses *hmSOD1* in nearly all tissues, including neurons, and the *hmSOD1 C. elegan* models controlled by the muscle-specific promoter of *myo-3* largely express *hmSOD1* among all muscles besides the pharynx.

The universal expression of *hmSOD1* in *C. elegans* blocks natural biological responses to oxidative stress and induces the aggregation of toxic proteins, while the expression of *hmSOD1* in all *C. elegans* neural systems leads to motor defects and neuron transmission damage in *C. elegans* models. The establishment of a *SOD1* single copy (*A4V, H71Y, L84V, G93A*, and *G85R*) mutation knockout *C. elegans* model can analyze the unique effect of the special mutation on the degeneration of cholinergic and glutamic MNs in *C. elegans* models. These findings indicate that the pathogenesis of ALS is a neuronal subtype-specific gain of toxic functions, as well as a loss of physiological functions (Liguori et al., 2021). Moreover, loss of SOD1 function is the main cause of glutamate neuron degeneration after oxidative stress in *C. elegans* models, and the toxic functions gaining of SOD1 protein may cause cholinergic MNs degeneration in *C. elegans* models (Baskoylu et al., 2018). In the ALS-like *hmSOD1 C. elegans* model containing the ALS *G85R* mutation, a relatively high level of *hmSOD1* was expressed in the neurons of *C. elegans*, resulting in movement defects of *C. elegans* expressing *SOD1 G85R* (Baskoylu et al., 2018).

Although morphological abnormalities of neurons and discernible survival or behavioral changes were not observed in the *C. elegans* model of *hmSOD1* expression, the *hmSOD1 C. elegans* model demonstrated decreased resistance to paraquat-induced oxidative stress, significantly reducing the degradation ability of *hmSOD1* proteins, leading to the abnormal accumulation of *hmSOD1* proteins among muscular cells. Moreover, the final pathological phenotypes of *hmSOD1* proteins in the *C. elegans* models were similar to the pathologically altered features observed in the post-mortem tissues of ALS patients.

The transgenic *C. elegans* model of entire neuronal expression of *hSOD1 G85R* using the synaptobrevin (*snb-1*) gene promoter coupled with yellow fluorescent protein (YFP) exhibits serious movement defects and paralysis (Wang et al., 2019). The observed phenotypes in this *C. elegans* model correlated with abnormal intra-neuronal aggregation of the mutated *SOD1* protein. Other *hmSOD1* genetic *C. elegans* models expressing various *SOD1* mutants, such as *G85R, G93A*, and *127X*, can be used to directly compare the differences in the aggregation, toxicity, and cellular interactions of *hmSOD1* proteins induced by different *SOD1* mutants. The *hmSOD1-YFP C. elegans* models expressing the *SOD1 G85R, G93A*, and *127X* mutants in muscular cells using the muscle-specific promoter of *unc-54* gene did not exhibit significant cell dysfunction (Gidalevitz et al., 2009). When these *SOD1* mutations were transferred into the genetic background of temperature-sensitive mutations in *C. elegans*, the toxins produced by the mutated SOD1 protein were significantly enhanced, resulting in a variety of toxic phenotypes. Therefore, it was suggested that the *hmSOD1* produced toxic phenotypes that might not only be the aggregate toxic products of *hmSOD1* proteins but are also closely associated with genetic interactions in the genetic background.

The *C. elegans* body locomotion is controlled by excitatory (acetylcholine, glutamate) and inhibitory (GABA) neurons. Stable overexpression of *hmSOD1 G93A* mutation causes the degeneration of GABAergic MNs, whereas single-copy expression of *hmSOD1 G93A* mutation only causes oxidative stress-induced cholinergic and glutamic neurodegeneration (Braems et al., 2021). Endogenous *SOD1* mutations, such as *hmSOD1 G93A*, trigger glutamate neuronal death in *C. elegans* (Baskoylu et al., 2018).

Metformin increased *daf-1* and *lgg-1* gene expression in *hmSOD1 C. elegans* model. The effects of Metformin on the lifespan of *hmSOD1 C. elegans* models were modulated by *daf-16* and *lgg-1* genes. In *lgg-1* deficient *C. elegans*, metformin did not restore the movement, amount, or morphology of MNs (Senchuk et al., 2018). Metformin significantly prolonged the lifespan, improved exercise performance, and enhanced the antioxidant activity of *hmSOD1 C. elegans*, further indicating that metformin enhanced the expression of *lgg-1, daf-16, skn-1*, and other genes that regulate autophagy, longevity, and oxidative stress in *hmSOD1 C. elegans* models. Therefore, *lgg-1* or *daf-16* overexpression alleviates aging and pathological abnormalities in *hmSOD1 C. elegans* models, whereas genetic deletion of *lgg-1* or *daf-16* eliminates the beneficial effect of metformin in *hmSOD1 C. elegans* models. The median survival period of *lgg-1* overexpression of *hmSOD1*^*G*93*A*^ *C. elegans* increased relative to *hmSOD1*^*G*93*A*^ *C. elegans*, indicating that activation of the *lgg-1* gene reduces the neurotoxicity induced by *hmSOD1* protein aggregation by inducing *hmSOD1* protein degradation, thereby promoting the lifespan of *hmSOD1 C. elegans*. Although both *lgg-1* and *daf-16* extend the *hmSOD1 C. elegans*, no additive effects are observed when these two genes are simultaneously overexpressed (Alberti et al., 2010). A regulatory factor, ubiquitin-specific processing protease 7 (USP7), affects the elimination of misfolded proteins such as hmSOD1 and TDP-43. The USP7 effect is modulated by both the transforming growth factor β and the small mother against decapentaplegic (SMAD) pathways, is mediated by USP7-NEDD4L-SMAD2 axis, and its activation promotes the protein quality control. Furthermore, Math-33 deletion inhibited protein toxicity induced by *hmSOD1 G85R* mutation in the *hmSOD1 G85R C. elegans* model (Zhang et al., 2020).

### 5.2 *C. elegans* carrying *TDP-43* mutations

The *TDP-43* transgenic *C. elegans* models were reproduced by expressing wild-type human *TDP-43* (hTDP-43^WT^) protein in neurons to study TDP-43 functional alterations and neurotoxicity in the pathogenesis of ALS (Ash et al., 2010). *TDP-43* transgenic *C. elegans* models were generated using *snb-1* gene promoters to drive human *TDP-4*3 (*hTDP-4*3) cDNA expression in whole neurons. This *TDP-43* transgenic *C. elegans* model exhibited distinctive uncoordinated phenotypes, such as slow movement and inappropriate responses to stimuli. The *C. elegans* model first exhibits these phenotypes at the larval stage and constantly retains these phenotypes throughout adulthood. This *C. elegans* model indicated that these uncoordinated phenotypes correlated with the synaptic lesions of abnormal MNs. The mechanism by which abnormal nuclear TDP-43 activity leads to abnormal synapses is not yet known, but it indicates that excessive activation of the nuclear TDP-43 protein might change some components of RNA metabolism, such as alternative RNA splicing, resulting in the dysfunction of some special proteins necessary for normal synapse functions.

*TDP-43* transgenic *C. elegans* models show that TDP-43 dysfunction results in insufficient chromatin processing and dsRNA accumulation, which may activate natural immunological systems and promote neuroinflammation (Milstead et al., 2023). It is currently understood that the neurodegenerative disease-related proteins, especially TDP-43 (ALS), amyloid beta and *tau* (Alzheimer's disease), α-synuclein (Parkinson's disease, PD), and FUS (frontotemporal dementia, FTD), partially show some characteristics of misfolding prion proteins. In addition, the *TDP-43 C. elegans* model also showed that the autophagy-lysosome pathway is the prion-like transmission pathway of *TDP-43* protein (Sandhof et al., 2020). Meanwhile, it was discovered that the regulation of autophagy or exocytosis/endocytosis can affect the transfer of these misfolded proteins (Tyson et al., 2017). The TDP-43 protein is an intracellular protein but can promote prion-like transmission of TDP-43 by entering the lysosomal system in the *TDP-43 C. elegans* model (Peng et al., 2020). This suggests that the autophagy-lysosome system and endocytosis-dependent endolysosomal pathway are related to the elimination of TDP-43 protein aggregates (Liu et al., 2017).

The currently developed *TDP-43* transgenic *C. elegans* model also showed some similar phenotypes in that normal *hTDP-43*^*WT*^ overexpression in all neurons caused different scales of movement defects (Liachko et al., 2010). Moreover, the overexpression of many human ALS-related *TDP-43* mutations, such as *G290A, A315T*, and *M337V*, causes a series of movement dysfunction phenotypes similar to those in ALS patients. It also indicates that these phenotypes of motor dysfunction worsen over time. The above-described similar phenotypes reproduced by the mutant *TDP-43* transgenic *C. elegans* models reproduce major features seen in ALS patients, such as progressive muscle weakness and atrophy, shortened life span, and MNs degeneration accompanied by both hyperphosphorylation, truncation, and ubiquitination of TDP-43 proteins and the accumulation of detergent-insoluble TDP-43 proteins. The *TDP-43 C. elegans* model provides an ideal *in vivo* model for exploring the cellular and molecular mechanisms underlying ALS. These *TDP-43 C. elegans* models may be used to search for potential alterations in TDP-43 function in the pathogenesis of ALS, possibly revealing the neurotoxic mechanisms of TDP-43 associated with ALS, and ultimately finding possible targets for developing novel therapeutic measures.

Twenty-four human homologous genes that inhibit *TDP-43* motor dysfunction after a reduction in RNAi-targeted gene expression have been identified. These genes involve multiple pathways, including the energy production and metabolism genes *cox-10, F23F12.3, F55G1.5, paqr-1*, and *cox-6A*, the extracellular matrix and cytoskeleton genes *col-89, glie-8, hs-5, sax-2, vab-9*, and *zig3*, the ion transport genes *cnnm-3, C13B4.1*, and *unc-77*, the nucleic acid function genes *dna-2, tbx-11*, and *umps-1*, protein balance genes *C47E12.3, gpx7*, and *pcp-5* and the signal transduction genes *F31E3.2, F40B5.2*, and *Y44E3A4* (Liachko et al., 2019). Human *TDP-43* and *C. elegans TDP-1* are functional homologs with similar RNA-binding activity, conserved N-terminus, nuclear localization signal (NLS), and RNA recognition motifs (Mitra et al., 2019). The *C. elegans* models of human *TDP-43* or *C. elegans TDP-1* expression in entire neurons produce the uncoordinated, slow, and GABAergic MNs constriction. In addition, the *C. elegans* model of targeted mutant *TDP-43* expression in *C. elegans* GABAergic MNs can lead to age-dependent progressive paralysis, GABAergic neuronal degeneration, and synaptic lesions. Mutated exogenous *TDP-43* induces oxidative stress and aberrant expression of endogenous *TDP-1* decreases neuronal function and lifespan in *C. elegans models*. Excessive phosphorylation of TDP-43 can interfere with protein homeostasis, leading to neurotoxicity in *C. elegans* model. Among the kinases involved, cyclin-dependent kinase 7 is directly involved in TDP-43 phosphorylation and decreases TDP-43 phosphorylation by inhibiting the produce of this enzyme in *C. elegans* model (Liguori et al., 2021). *TDP-1* deficiency or deletion leads to dsRNA accumulation and enhances exogenous RNAi by increasing nuclear RNAi in *C. elegans*. *C. elegans* nuclear RNAi involves chromatin alterations that are regulated by heterochromatin protein like-2 (HPL-2) and the homolog of heterochromatin protein 1 (HP1). TDP-1 interacts directly with HPL-2, and the TDP-1 deletion significantly changed the chromatin association of HPL-2. These molecular changes, replicated in *C. elegans* models, indicate that the nuclear depletion of *hTDP-4*3 might result in alterations in disease-related RNA metabolism during the pathogenesis of ALS (Saldi et al., 2018).

### 5.3 *C. elegans* carrying *FUS* mutations

*FUS* is involved in various functions associated with DNA and RNA processing (Birsa et al., 2020). Prominent features of the mutant *FUS C. elegans* model include cytoplasmic mislocalization, generation of FUS aggregates, age-dependent motor defects, paralysis, and impaired GABAergic neurotransmission. In addition, the transmission of MNs to muscle tissues is reduced through ultrastructural and electrophysiological methods, indicating a role for FUS in synaptic vesicle tissue and nerve transmission (Braems et al., 2021). To establish the ALS-*FUS C. elegans* model, the *FUS R524S* and *P525L* allele mutations are inserted into the c-terminus NLS of endogenous *fust-1* gene in chromosome 2 to generate an ALS-*FUS* animal model. Overexpression of *FUS* leads to severe motor defects and damages neurons and muscle autophagy without damaging the neuronal ubiquitin-proteasome pathway in *FUS C. elegans models* (Baskoylu et al., 2022). *FUS C. elegans models* have also shown that autophagy dysfunction may contribute to the development of ALS. However, the mode and degree of autophagy dysfunction in ALS pathogenesis are not fully understood (Chua et al., 2022). Excessive expression of *FUS* in *C. elegans models* affects the self-regulation of *FUS*, thereby affecting RNA processing, stability, and protein homeostasis. The irreversible hydrogel formed by the mutant *FUS* gene impairs the function of heterogeneous ribonucleoprotein particles, reducing the speed of new protein synthesis in mutant *FUS C. elegans models* (Markert et al., 2020). Disruption of these functions occurs by negatively affecting autophagy. The steady-state level of FUS is crucial for normal neuronal function, as increased FUS levels accelerate diseases such as ALS (Zhang et al., 2018b).

### 5.4 *C. elegans* carrying *C9ORF72* mutations

The mutative *C9ORF72* gene results in the loss of its functions, gain of toxic functions, or both; however, whether these *C9ORF72 dysfunctions* are involved in the pathogenesis of ALS is not yet fully understood. It has been revealed that the *C9ORF72* pathogenic GGGGCC (G4P2) repeat mutation significantly decreases *C9ORF72* expression. The *C. elegans* model harboring the 3rd and 4th exon deletion in *C. elegans* ALS-associated gene homolog *alfa-1* led to a drastic reduction in *C9ORF72* expression, exhibiting a movement defect resulting in age-dependent paralysis and the degeneration of GABA-ergic MNs. Moreover, the mutant *alfa-1 C. elegans* model showed osmotic stress sensitivity to provoke MNs degeneration.

The causative G4P2 repeat among the *C9ORF72* gene is the key cause of familial and sporadic ALS found up to date. It is not known whether the *C9ORF72* functions are the same as those of other ALS-related genes such as *TDP-43* and *FUS*. It is important to note that *C9ORF72* expression is also reduced in many ALS cases with the negative pathogenic G4P2 repeat, suggesting that *C9ORF72* may exert synergistic effects with other ALS-related genes. In order to further understand the *C9ORF72* potential synergistic effects with other ALS-related genes, the *C. elegans* model carrying mutative *TDP-4*3 A315T or *FUS*ΔUS5 combined with the null *alfa-1* mutation were established, which showed that the null *alfa-1* expression decreases and the paralytic phenotypes exacerbates but does not change the toxic of mutative FUS protein, which suggests that the mutative *C9ORF72* gene exerts a synergistic effect with the mutative *TDP-43* gene in the same MNs toxic pathway but not the mutative *FUS* gene. Reduced *alfa-1/C9ORF72* expression enhanced the toxicity of the mutated TDP-43 protein but did not affect the FUS protein in the *C. elegans* model. This suggests that the genetic network is composed of many genes involved in the pathogenesis of ALS, where ALS-related genes interact each other based on the study in this *C. elegans* model. The model development of genetic networks in *C. elegans* is helpful for understanding the synergistic toxic mechanisms of ALS-related genes in neuronal loss in ALS (Therrien and Parker, 2014a).

*C. elegans* models studying the mechanism associated with the gain or loss of *C9ORF72* gene functions revealed that the deletion of *C9ORF7*2 gene leads to early serious paralysis, nuclear transport impairment, lysosomal homeostasis dysregulation, neurodegeneration, and neuronal death. The *C9ORF7*2 *C. elegans* model can be used to investigate endocytosis, which reveals that *alfa-1* deletion causes a defect in lysosome homeostasis, such as the dysfunction of lysosome reformate and the degradation of endocytosal elements; thus, the *C9ORF7*2 *C. elegans* model is the first chosen model that is used to identify whether or not *C9ORF72* is involved in the endocytosis pathway (Therrien and Parker, 2014b).

Partial neurodegenerative diseases have common or overlapping pathogenic mechanisms at the cellular level, such as protein misfolding, excitotoxicity, and altered RNA homeostasis. Moreover, genetic factors contributing to common, cross, or overlapping pathogenesis imply the existence of a possible genetic network of neurodegeneration in the pathogenesis of ALS. The recently discovered overlapping pathogenic mutation *C9ORF72* in both ALS and FTD is perhaps the best illustration. It was thought that the different neurodegenerative diseases were distinct entities and possessed a single genetic spectrum; however, the current study findings are changing this opinion, considering that some neurodegenerative diseases have a similar genetic spectrum. Following the progression of the related genetic discovery, there is an urgent need to establish a novel genetic model to not only investigate the causative mechanisms associated with the pathogenic mutations of neurodegeneration but also to study the possible genetic interactions among the different mutated genes, which may reveal novel pathogenic targets. Based on the evolutionary conservation of various pathogenic genes with *C. elegans, the C. elegans* genetic models may be the first candidates to study the genetic interactions among these pathogenic genes; therefore, *C. elegans* genetic models, such as the mutative *C9ORF72* gene, may be used to model both ALS and FTD to study the potential genetic interactions and networks in the pathogenesis of ALS and FTD (Therrien and Parker, 2014b).

## 6 Conclusions and future perspectives

Establishing invertebrate models provides new approaches and tools for a better understanding of ALS (Tables 1–3). Various transgenic invertebrate models have been widely used to explore ALS, especially fALS. Transgenic invertebrate models are not ideal for studying sporadic ALS or searching for drugs to cure and/or prevent ALS. Novel evidence from the experimental results of small animal models, such as invertebrate animals, to human clinical trials is very limited and challenging in the investigation of ALS because of the significant differences in the genomes, molecules, and anatomy between invertebrate animals and humans. For example, some invertebrate genetic models display mild ALS phenotypes without neurodegeneration. The shorter lifetime of most invertebrates is not long enough to induce the typical neurodegeneration that occurs in humans. In addition, only a small portion of alternative splicing transcription exons is conserved between humans and invertebrates. These differences between invertebrates and humans might result in substantial differences between the results from invertebrates and humans for ALS, such as pathogenesis, pathology, and medical treatment (Zhu et al., 2023). Of course, the large animal models such as vertebrates, monkeys, and orangutans have smaller species differences in neuropathology. These are the optimal models for studying the pathogenesis, pathology, and treatment of human diseases. However, there are also many disadvantages such as high cost, gene targeting inefficiency, the limitations of resources and ethics, and longer time, which are not beneficial for the extensive use of large animal models (Zhu et al., 2023). The combining application of invertebrate and vertebrate animal models can better mimic ALS and is beneficial for detecting potential causative pathways and effective measures to cure and/or prevent ALS. Generally, a better understanding of the advantages and limitations of different animal models will enable better use of different animal models to mimic ALS. In the future, the development of new invertebrate models will focus on establishing invertebrate models with different mechanisms, including genetics, which will hopefully reveal novel evidence in the pathogenesis and etiology of ALS and reveal for novel strategies to cure or prevent ALS (Myszczynska and Ferraiuolo, 2016).

**Table 1:** Comparison of invertebrate genetic models of ALS: yeast.

**Groups**	**Type**	**Characterization**	**Phenotype**	**Influence**	**References**
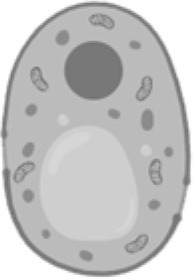 Yeast genetic models	**SOD1**	**Produced by** • SOD1 mutations (A4V/G37R/G93A/H48Q/S134N)	**Gain-of-function toxicity** (1) Reducing cell viability without forming insoluble protein aggregates	None	(Rabizadeh et al., 1995; Kryndushkin et al., 2012; Bonifacino et al., 2021)
	**Prion-like proteins**	**Produced by** TDP-43 • hTDP-43^WT^ • TDP-43 mutations (M337V/N345K/N390D/Q331K/Q343R/R361S) FUS • hFUS^WT^ • FUS mutations Other • hnRNPA2 • TAF15 • EWS	**Gain-of-function toxicity** (1) Inhibits cell growth (2) Deranges morphology (3) Generate cytotoxicity: leads to cell death	None	(Bonifacino et al., 2021)

**Table 2:** Comparison of invertebrate genetic models of ALS: *Drosophila melanogaster*.

**Groups**	**Type**	**Characterization**	**Phenotype**	**Influence**	**Reference**
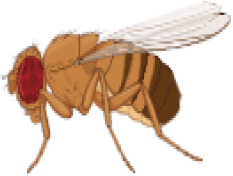 *Drosophila melanogaster* genetic models	**SOD1**	**Produced by**• hSOD1^WT^ • SOD1mutations (A4V/G37R/G85R/H48R/H71Y)	**hSOD1 mutations (G85R/H48R/H71Y) in endogenous dSOD1** (1) Leads to reduced survival, developmental defects, and dyskinesia **Only hSOD1**^**WT**^ **in motor neurons**(1) Prolong lifespan: without affecting locomotion or motor neuron survival(2) Does not prevent age-related movement disorders**hSOD1**^**WT**^ **and hSOD1 mutations (A4V/G85R)**(1) Electrophysiological defects(2) The SOD1 abnormal accumulation (3) The stress response surrounding glial cells(4) Cellular (motor neurons) and non-cellular (glial cells) autonomic damage• Manifested as: the SOD1 toxicity propagate from one cell to another, induces progressive motor dysfunctions	(1) Antioxidant compounds: • Improve exercise performance • Prolong lifespan • Reduce SOD1 cytoplasmic inclusion bodies	(Parkes et al., 1998; Clement et al., 2003; Boillée et al., 2006; Watson et al., 2008; Walters et al., 2019; Braems et al., 2021; Layalle et al., 2021; Liguori et al., 2021)
	**TDP-43**	**Produced by** • Endogenous dTDP • hTDP-43^WT^ • TDP-43 mutations (A315GOF)	**Endogenous dTDP** (1) Lack of dTDP: • Appears externally normal • But presents the deficiency of locomotion behaviors, the reduction of life span, the anatomical defects of NMJs, and the decrease of dendrite branches (2) Overexpression of dTDP: • Premature lethality • Neuronal loss • The defects of NMJs • Locomotor deficits**hTDP-43**^**WT**^**, hTDP-43 mutations and TBPH** (1) Overexpression of hTDP-43^WT^ and TBPH: • affects lifespan, mobility, axonal transport, and pupal shell sealing (2) Depletion of TBPH: • Leads to movement disorders and shortened lifespan (3) Overexpression of hTDP-43: • Induces mitochondrial dysfunction • Inhibits mitochondrial complex I activity • Highly fragmented mitochondria in the axons of motor neurons**Metabolic dysfunction** (1) Glucose metabolism: • Increased Pyruvic acid • Increased the mRNA level of PFK(2) Lipid metabolism: • Reduced carnitine shuttle and β oxidation	(1) Gene modification of TDP-43: • Upregulation of Pab1-binding protein 1: enhances the TDP-43 toxicity and result in more severe TDP-43 induced phenotypes • Overexpression of ubiquilin 1: enhances the severity of TDP-43 phenotypes • Co-expression of VCP: induced TDP-43 phenotypes (2) The PTK2-TBK1-SQSTM1 axis: • PTK2 inhibition: significantly reduced the ubiquitin aggregate and reduced the cytotoxicity in the TARDBP-induced protein disease • The non-phosphorylated form of SQSTM1: significantly inhibited the accumulation and neurotoxicity of insoluble polyubiquitin proteins (3) NBs, Mp, CG5445 and NEAT1-1: • alleviated the cytotoxicity of TDP-43 Drosophila neurons and improved motor or eye symptoms (4) Gemin3: • Aggravates vitality defects, motor dysfunction, and muscle atrophy while inhibiting the overgrowth of the NMJ (5) Knocking down the 3-fold lethal [Su (Tpl)] gene inhibitor: • Inhibits the morphological defects of compound eye and medial retina	(Feiguin et al., 2009; Kim et al., 2009; Lu et al., 2009; Elden et al., 2010; Hanson et al., 2010; Li et al., 2010; Ritson et al., 2010; Voigt et al., 2010; Estes et al., 2011; Lin et al., 2011; Miguel et al., 2011; Maccioni et al., 2018; Uechi et al., 2018, p. 3; Zhang et al., 2018a; Cacciottolo et al., 2019; Manzo et al., 2019; Walters et al., 2019; Lee et al., 2020; Wang et al., 2020; Layalle et al., 2021; Liguori et al., 2021; Matsukawa et al., 2021)
	**FUS/TLS**	**Produced by** • hFUS/TLS drosophila orthodox • hFUS/TLS^WT^ • FUS/TLS mutations (R518K/R521C/R521H)	**hFUS/TLS mutations (R518K/R521C/R521H)**(1) Severe neurodegeneration in Drosophila eyes(2) Locomotor dysfunction and premature lethality (3) Synaptic damage • Manifested as: progressive age-dependent neuronal degeneration **hFUS/TLS drosophila orthodox (Cabeza) deficient** (1) Reduced lifespan and the locomotor deficits **hFUS/TLS**^**WT**^ (1) Only resulted in very mild eye degeneration	(1) Associate with TDP-43: • Both FUS/TLS and TDP-43 proteins exert some damaged effect together *in vivo* in the pathogenesis of ALS (2) Cabeza: • Functional loss are fatal: only a few develop into adulthood, with severely shortened lifespan and motor disorders • Expressing Caz: can repair climbing and flight defects • Overexpressing Caz/FUSWT exhibit opposite NMJ electrophysiological phenotypes (3) Inhibiting nuclear output of FUS: • Chameau and NAA60: inhibit the rough eye phenotype induced by caz knockdown • XPO1 and NUP 154: down-regulation prevents the toxicity induced by FUS (4) Kap-β2: • Inhibits PY-NLS and reverses FUS fibrosis • Rescues degeneration caused by FUS-ALS	(Chen et al., 2011; Lanson et al., 2011; Guo et al., 2018; Braems et al., 2021; Yamaguchi et al., 2021)

**Table 3:** Comparison of invertebrate genetic models of ALS: *Caenorhabditis elegans*.

**Groups**	**Type**	**Characterization**	**Phenotype**	**Influence**	**Reference**
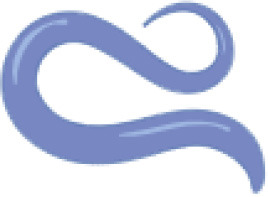 *Caenorhabditis elegans* genetic models	**SOD1**	**First established** • 2001 **Mutations expressed in** • By HSP16-2 promoter: almost in all tissues including neurons • By myo-3 muscle-specific promoter: in all muscle tissues besides pharynx **Produced by** • hSOD1^WT^ • SOD1 mutations (A4V/G37R/G85R/G93A/H71Y/L84V)	**Neuron subtype-specific** (1) Increased toxic function: leads to the degeneration of cholinergic neurons (2) Loss of function: leads to the degeneration of glutamatergic neurons • Manifested as: motor defects and neuronal transmission damage **Not observe** • Morphological abnormalities • Discernable survival • Behavior changes **Motor dysfunction** (1) SOD1-G93A: leads to the degeneration of GABAergic motor neurons (2) CRISPR/cas9 - endogenous SOD-1: leads to cholinergic and glutamatergic neurodegeneration	(1) snb-1/G85RSOD1-YFP: • Resulting in serious locomotor defects and paralysis (2) unc-85/SOD1(G85R/G93A/127X)-YFP: • resulting in mild cellular dysfunction (3) Genetic background: • Temperature-sensitive mutations enhanced the toxicity and produced a variety of toxic phenotypes (4) Metformin: • Prolonged the lifespan • Improved exercise performance, and • Enhanced antioxidant activity	(Oeda et al., 2001; Gidalevitz et al., 2009; Wang et al., 2009; Baskoylu et al., 2018; Senchuk et al., 2018; Braems et al., 2021; Liguori et al., 2021)
	**TDP-43**	**Mutations expressed in** • By snb-1 promoter: among all neurons **Produced by** • hTDP-43^WT^ • TDP-43 mutations (A315T/G290A/M337V)	**Distinctive uncoordinated phenotypes** (1) Non-sinusoidal, slow movement, and inappropriate responses to stimulus • First exhibits at the larval stages • Remains throughout its adulthood **TDP-43 dysfunction** (1) Insufficient Chromatin processing (2) Accumulation of dsRNA • Manifested as: activate the innate immune system and promote neuroinflammation **Motor dysfunction** (1) hTDP-43^WT^: causes the different scales of defects of motor (2) TDP-43 mutations (A315T/G290A/M337V): causes a series of similar motor dysfunction phenotype with ALS • Manifested as: muscle progressive paralysis, the shortened lifespan, and motor neurons degeneration	(1) hTDP-43 or *C. elegans* TDP-1 panneuronal expression: • Produce worms with uncoordinated, slow motion and GABAergic motor neuron constriction (2) Exogenous TDP-43: • Mutated: induces oxidative stress, abnormal expression of endogenous TDP-1, and decreased neuronal function and lifespan • Excessive phosphorylation: interferes with protein homeostasis, leading to neurotoxicity (3) *C. elegans* TDP-1: • Deleting TDP-1: leads to the accumulation of dsRNA, and significantly changed the Chromatin association of HPL-2 • Deleting tdp-1 gene: leads to the enhancement of RNAi	(Ash et al., 2010; Liachko et al., 2010; Saldi et al., 2018; Mitra et al., 2019; Milstead et al., 2023)
	**FUS**	**Produced by** • FUS mutations (R524S/P525L)	**Damage neurons and muscle autophagy** (1) Cytoplasmic mislocalization (2) The formation of FUS aggregates (3) Age-dependent motor deficits (4) Paralysis (5) Impaired GABA-ergic neurotransmission • Manifested as: severe motor defects **Affects the regulation of the FUS itself** (1) RNA processing and stability (2) Protein homeostasis		(Markert et al., 2020; Braems et al., 2021)
	**C9ORF72**	**Produced by** • C9ORF72 gene deletion • C9ORF72 pathogenic G4P2 repeat mutation • C9ORF72 gene homolog alfa-1 mutation or deletion	**C9ORF72 gene deletion** (1) Early severe paralysis (2) Nuclear transport impairement (3) Lysosomal homeostasis dysregulation (4) Neurodegenration (5) Neuron death **C9ORF72 pathogenic G4P2 repeat mutation** (1) Significantly decrease the C9ORF72 expression **C9ORF72 gene homolog alfa-1** (1) Alfa-1 mutation: the third and fourth exons deletion • leads to the drastic reduction of C9ORF72 expression • Motility defects • Stress sensitivity (2) Alfa-1 deletion • Lysosomal homeostasis defects: including dysfunctions in lysosomal reformation and the degradation of endocytosed elements	**Synergistic effects with other ALS-related genes** (1) Mutant TDP-43 A315T combined with the null mutation of alfa-1: • Alfa-1 expression decreases • Enhances the toxicity of mutant TDP-43 protein • Exacerbates the paralysis phenotype (2) Mutant FUS US5 combined with the null mutation of alfa-1: • Unenhanced the toxicity of mutant FUS proteins	(Therrien and Parker, 2014a,b)

There are advantages of using invertebrate models over zebrafish, mice, and induced pluripotent stem cell (iPSC) models in ALS studies ALS is an aggressive and fatal degenerative disease that damages the nervous system. To study the pathogenesis of ALS, a series of *in vivo* and *in vitro* models, including yeast, flies, worms, zebrafish, mice, and human iPSCs from patients with ALS, have been established one after another. Although mouse models and invertebrate model zebrafish are the major small-animal models of ALS, the discovery of iPSCs provides a novel opportunity to study the molecular phenotypes of ALS within human cells. It is important that iPSC technology can model both familial and sporadic ALS in relevant human genetic backgrounds as well as research drug development and the potential pathogenesis of ALS through personalized or targeted iPSC-intervening mice and zebrafish models. Further identification of iPSCs using mouse and zebrafish models might still be necessary to determine which phenotypes, responsible genes, and putative target compounds most likely reflect upstream disease driving, as opposed to epiphenomenon-related or even compensatory mechanisms (Hawrot et al., 2020). In general, invertebrate zebrafish, vertebrate mice, and iPSCs models are complementary to ALS studies.

There are also advantages of using *in vivo* over *in vitro* studies. Using *in vivo* models such as animal models or human trials allows for a better simulation of the complexity of diseases, including their etiology, progression, and response to treatment. In contrast, *in vitro* studies typically provide limited information and struggle to capture the intricate changes that occur throughout the disease process. *In vitro* studies are often conducted under simplified conditions, making it challenging to replicate diverse interactions within living organisms. This may lead to inaccuracies in the results when compared with the *in vivo* situation (Myszczynska and Ferraiuolo, 2016; Gois et al., 2020).

Maintaining physiological relevance can be a challenge in *in vitro* studies, which require adjustments to align with the physiological conditions found in living organisms. However, complete replication may not be possible. In contrast, *in vivo* research maintains higher biological complexity throughout the organism. The *in vivo* environment encompasses cells, tissues, organs, and their interactions, providing a realistic representation of biological phenomena (Myszczynska and Ferraiuolo, 2016; Gois et al., 2020).

A rich network of interactions between different cells and organs within living organisms is crucial for studying the comprehensive impact of diseases. *In vivo* models capture the dynamics of these networks. *In vitro* studies may struggle to accurately replicate the complex interactions between cells and organs, limiting our understanding of the overall biological system. Although *in vitro* studies offer insights into cellular responses, their limitations are evident when assessing the effects of drugs on an entire living organism. In this context, *in vivo* research provides a more direct assessment of the effects of drugs on the overall biological system, thereby enhancing the accuracy of predicting potential therapeutic efficacy and side effects (Myszczynska and Ferraiuolo, 2016; Gois et al., 2020; Bonifacino et al., 2021).

Optogenetic strategies are promising candidates for exploring the use of invertebrate models to develop novel genetic models to investigate the pathological mechanisms of ALS. Optogenetics is a novel advanced technology used to identify potential therapeutic strategies that are achieved by regulating ion flow and electrical signals in neuronal cells and neural circuits damaged by disease. At present, optogenetics has not been extensively applied to animal models of neurodegenerative diseases such as ALS and PD. Recently, human and *D. melanogaster* receptors were activated by light and rearranged during transfection, creating one-component optogenetic tools called Opto-hRET and Opto-dRET. These rearranged receptors strongly induced the MAPK/ERK proliferative signaling pathway in cells cultured under blue light stimulation. Light activation of Opto-dRET suppressed mitochondrial defects, tissue degeneration, and behavioral deficits in PINK1B9 *D. melanogaster* of a familial PD-associated kinase, PTEN-induced putative kinase 1 (PINK1) loss. Mitochondrial fragmentation was rescued by applying Opto-dRET through the PI3K/NF-κB pathway in human cells with a loss of PINK1 function. This optogenetic evidence demonstrated that a light-activated receptor can ameliorate disease hallmarks in a genetic model of PD. Similarly, optogenetic methods may be used in ALS models, including *in vivo* and *in vitro* models, to study the pathogenesis, pathology, and potential treatments for ALS. The use of invertebrate models to develop novel genetic and optogenetic strategies to investigate the pathological mechanisms of ALS is a promising research tool (Ingles-Prieto et al., 2021).

## Author contributions

LZ: Writing—original draft, Writing—review & editing. RX: Conceptualization, Funding acquisition, Writing—original draft, Writing—review & editing.
